# Differential training benefits and motor unit remodeling in wrist force precision tasks following high and low load blood flow restriction exercises under volume-matched conditions

**DOI:** 10.1186/s12984-024-01419-5

**Published:** 2024-07-19

**Authors:** Yen-Ting Lin, Chun-Man Wong, Yi-Ching Chen, Yueh Chen, Ing-Shiou Hwang

**Affiliations:** 1 Department of Ball Sport, National Taiwan University of Sport, Taichung City, Taiwan; 2 Department of Physical Therapy, College of Medicine, National Cheng Kung University, Tainan City, Taiwan; 3 Department of Physical Therapy, College of Medical Science and Technology, Chung Shan Medical University, Taichung City, Taiwan; 4 Orthopedic Department, Kaohsiung Veterans General Hospital Tainan Branch, Tainan City, Taiwan; 5 Institute of Allied Health Sciences, College of Medicine, National Cheng Kung University, Tainan City, 701 Taiwan

**Keywords:** Hypoxia, Training load, Force regulation, Motor unit, Electromyography

## Abstract

**Background:**

Blood flow restriction (BFR) resistance training has demonstrated efficacy in promoting strength gains beneficial for rehabilitation. Yet, the distinct functional advantages of BFR strength training using high-load and low-load protocols remain unclear. This study explored the behavioral and neurophysiological mechanisms that explain the differing effects after volume-matched high-load and low-load BFR training.

**Methods:**

Twenty-eight healthy participants were randomly assigned to the high-load blood flow restriction (BFR-HL, *n* = 14) and low-load blood flow restriction (BFR-LL, *n* = 14) groups. They underwent 3 weeks of BFR training for isometric wrist extension at intensities of 25% or 75% of maximal voluntary contraction (MVC) with matched training volume. Pre- and post-tests included MVC and trapezoidal force-tracking tests (0–75%–0% MVC) with multi-channel surface electromyography (EMG) from the extensor digitorum.

**Results:**

The BFR-HL group exhibited a greater strength gain than that of the BFR-LL group after training (BFR_HL: 26.96 ± 16.33% vs. BFR_LL: 11.16 ± 15.34%)(*p* = 0.020). However, only the BFR-LL group showed improvement in force steadiness for tracking performance in the post-test (*p* = 0.004), indicated by a smaller normalized change in force fluctuations compared to the BFR-HL group (*p* = 0.048). After training, the BFR-HL group activated motor units (MUs) with higher recruitment thresholds (*p* < 0.001) and longer inter-spike intervals (*p* = 0.002), contrary to the BFR-LL group, who activated MUs with lower recruitment thresholds (*p* < 0.001) and shorter inter-spike intervals (*p* < 0.001) during force-tracking. The discharge variability (*p* < 0.003) and common drive index (*p* < 0.002) of MUs were consistently reduced with training for the two groups.

**Conclusions:**

BFR-HL training led to greater strength gains, while BFR-LL training better improved force precision control due to activation of MUs with lower recruitment thresholds and higher discharge rates.

## Background

Blood flow restriction (BFR) training has gained attention as an effective method for enhancing muscle strength and endurance using low-load resistance exercises [1, 2]. This training involves applying a specialized cuff or band to the proximal part of a trained limb, resulting in occlusion pressure that restricts arterial inflow and venous outflow during exercise [3, 4]. Compared to traditional resistance training, BFR training enhances mechanical tension through cell swelling and metabolic stress involving lactate and reactive oxygen species (ROS) [4, 5]. This enhancement of mechanical tension contributes to muscle hypertrophy and protein synthesis by activating several cellular signaling pathways such as the mammalian target of rapamycin (mTOR) and/or the MAPK [mitogen-activated protein kinase] pathways [6]. Metabolic stress in a hypoxic condition adds to the post-exercise release of hormones (such as growth hormone and IGF-1) that promote muscle hypertrophy and strength gains [7]. Despite the acknowledged facilitatory role of BFR in enhancing strength gains with low loads, there is an ongoing debate about the optimization of BFR application, such as the use of higher resistance loads for BFR training [8, 9].

The effects of traditional resistance training vary with the load used. High-load training, typically above 75% of one’s one-repetition maximum (1RM) [10], primarily aims to enhance muscle power and maximal strength. Low-load training, roughly 30–60% of 1RM (or MVC), is recommended for improving muscle endurance [11]. These protocols trigger distinct neuromuscular adaptations and motor unit (MU) recruitment strategies. High-load training tends to recruit a greater number of MUs of higher recruitment thresholds, promoting higher discharge rates and synchronization of MUs during muscle contractions. In contrast, low-load training favors the recruitment of slow-twitch, fatigue-resistant fibers, which have lower recruitment thresholds [12–14]. Interestingly, along with a superior strength gain, high-load resistance training could induce a greater increase than low-load resistance training in the efficiency of muscle activation to produce the same relative submaximal torques with lower EMG amplitude [15]. However, traditional high-load training may not necessarily contribute positively to force precision control. Bellew [2002] reported that older adults who received 12 weeks of high-intensity training on the quadriceps exhibited a significant increase in muscle strength, while force precision control under sub-maximal force levels remained unchanged after training [16]. In fact, strength-trained individuals often demonstrate larger force variability than do skill-trained individuals [18], as high-load training is associated with stronger MU synchrony [17] and larger inter-spike intervals [slower firing rates] of active MUs [18].

By analogy, the effects of BFR training are expected to vary with the load used, but research in this area is limited. Biazon et al. (2019) compared traditional high-load (HL) training, high-load training with BFR (BFR-HL), and low-load training with BFR (BFR-LL) over 10 weeks of leg extension training [8]. They found that all three groups showed similar significant improvements in maximum dynamic strength (1-RM), muscle cross-sectional area, and pennation angle of the trained muscles. Muscle activation was higher in the HL and BFR-HL groups than in the BFR-LL group before and after training. May et al. (2022) reported that HL (70% 1RM) and LL (20% 1RM) + BFR training produced similar outcomes in muscle strength, protein growth markers, and muscle size after 7 weeks of training [19]. It is worth noting that training volume (total work performed) influences strength gains [20, 21]. Strength gains reported in combined BFR and HL or LL protocols in the previous works could be confounded by variations in the total training volume, which were not adequately controlled. Moreover, to date, adaptive changes in the activation strategy of MUs for BFR-HL and BFR-LL training remain unclear. Current evidence simply shows that BFR has an immediate effect on changes in MU behaviors, including increases in the recruitment of larger MUs and the firing rate of smaller MUs during low-intensity exercise [22].

The aim of this study was to investigate the varying training effects on force control and the underlying adaptive changes in motor unit behaviors following 3 weeks of high-load and low-load BFR resistance training at equivalent training volumes. Since the remodeling of motor units is influenced by resistance load [16–18] and the application of BFR [22], force precision control based on motor unit activation strategies can be interactively modified through BFR training with varying loads. Our hypotheses were: (1) High-load BFR training protocols would yield superior strength gains compared to low-load BFR training protocols; (2) Low-load BFR training protocols would yield superior force precision control compared to high-load BFR training protocols; (3) Motor unit (MU) activation strategies during force scaling differ with the load used in BFR resistance training, with lower recruitment thresholds, higher discharge rates, and lower MU synchrony enhancing force steadiness for the low-load protocol.

## Methods

### Participants

Twenty-eight healthy young adults (15 males and 13 females, age: 22.7 ± 1.7 years, body mass: 61.4 ± 15.4 kg, height: 166.7 ± 9.2 cm) with no regular exercise habits were recruited for the present study. The participants were randomly assigned to two groups: blood flow restriction with high-load (BFR-HL) (7 males and 7 females) or blood flow restriction with low-load (BFR-LL)(8 males and 6 females). None of the participants had engaged in strength training for at least 6 months or used supplements, anti-inflammatory medications, or anabolic steroids during the experiment. No participants had history of diagnosed hypertension (systolic blood pressure > 140/90 mmHg), cardiovascular or pulmonary diseases, neuromuscular disease, or musculo-skeletal disorders. The study protocol was approved by the Institutional Human Research Review Board (Jen-Ai Hospital Institutional Review Board, project number 111 − 43). All participants signed a consent form before the experiment.

### Experimental protocol and setup

**Fig. 1:**
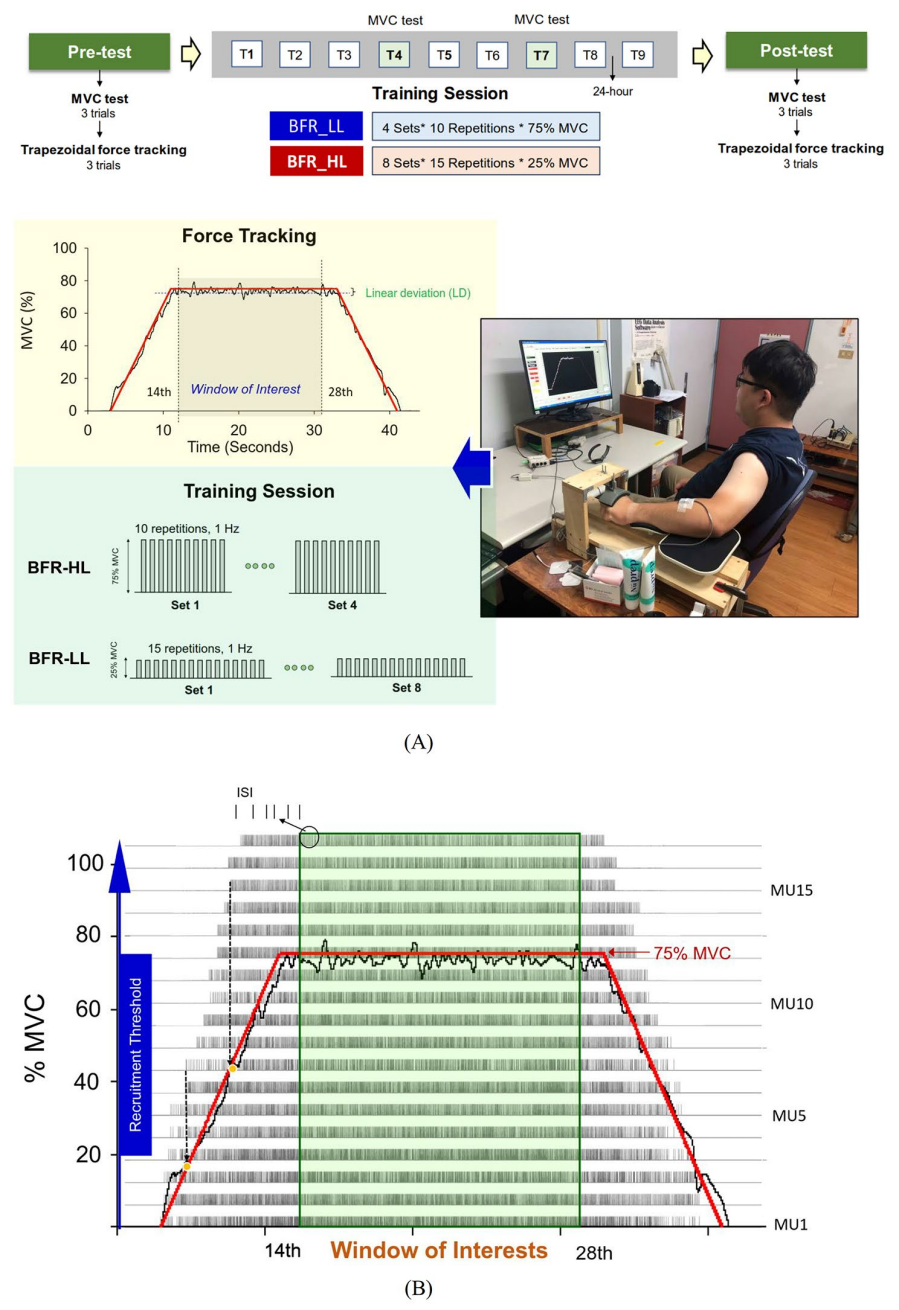
The experimental setup and sample data of force tracking. (**A**) Experimental flowchart and measurements for trapezoidal force tracking in the pre-test and post-test (top plot) and combined resistance exercise with blood flow restriction during wrist extension training (bottom plot). The mismatches between force trajectory and target line in the regions of interest were quantified using root mean square of force fluctuations and linear deviation (LD). During the training session, the BFR-HL and BFR-LL groups underwent resistance training with varying loads but equivalent training volumes (intensity*repetitions*set). The training volumes of each session for the BFR-HL and BFR-LL group were 75% MVC*10 repetitions*4 sets and 25% MVC*15 repetitions*8 sets. (**B**) Sample decomposition results of multi-channel surface EMG, resulting in motor unit spike trains. Recruitment thresholds for each identified motor unit were determined by aligning the force trajectory with the timing of the first discharge event. Discharge properties in the regions of interest, including mean and coefficient of variation (CV) of inter-spike interval, were also evaluated. (HL: high load; LL: low load)

In the initial laboratory visit, we collected anthropometric data and assessed the maximal voluntary contraction (MVC) of each participant. Subsequently, we measured the baseline capacity for force scaling using trapezoidal force tracking with visual feedback in the pre-test. Grouped into either BFR-HL or BFR-LL, participants underwent BFR training sessions across visits 1 to 9, with 24-hour gaps separating training visits. During the 4th and 7th visits, we re-evaluated MVC to adjust the training load for subsequent training and post-test force-tracking. The post-test was conducted on the 10th laboratory visit and involved the same measurements (MVC and trapezoidal force tracking) as the pre-test (Fig. 1(A)).

MVC of wrist extension was determined, when participants were seated with the forearm and palm positioned within a plastic splint on a wooden platform. The MVC of wrist extension was the peak value of three maximum contraction trials, separated by 3-minute rest periods. In the trapezoidal force-tracking task, instructions given to the volunteers were: ‘Please track the trapezoidal target-line displayed on the computer monitor by carefully adjusting your wrist extension force.’ The participants exerted isometric wrist extension force with their non-dominant limb to match a target signal displayed on a computer monitor (Fig. 1(A)). This target signal comprised an 11-second ramp-up phase, an 11-second ramp-down phase, and a 16-second static isometric contraction at 75% of MVC. The high target force was employed to aim at activating the full spectrum of trained motor units within the established decomposition limit (80% MVC) for utilization with the software of EMG Works version 4.3 (Delsys Inc., USA). Each tracking trial, including 3-second latent periods at the start and end, took 44 s to complete. During the pre-test and post-test, each subject performed 3 trials of the force-tracking task with at least 1 min of rest for recovery between trials. Force precision control (or force scaling) was assessed based on the performance of the static isometric contraction in the window of interest (14th–28th seconds). The force outputs of wrist extension during the MVC and trapezoidal isometric contraction were measured with a force transducer (Model: MB-100; Interface Inc., Scottsdale, AZ) and sampled at 1 kHz with an analog-to-digital converter with 16-bit resolution (DAQCard-6024E; National Instruments Inc., Austin, TX) using a custom LabVIEW program (National Instruments Inc., Austin, TX, USA). Concurrently with the force signals, we employed a multi-electrode EMG system comprising a sensor array (Bagnoli sEMG system; Delsys Inc., Natick, MA) to capture muscle activity in the extensor digitorum. The EMG sensor array consisted of five pins (each with a diameter of 0.5 mm) arranged in a 5 * 5 mm square configuration. This array was positioned along a line extending from the lateral epicondyle of the elbow to the second metacarpal bone, situated at a distance of 50–70 mm from the lateral epicondyle. Pairwise differentiation of the five pin electrodes generated four channels of surface EMG. These signals were subjected to amplification (gain: 1000) and band-pass filtering (cutoff frequencies: 20 and 450 Hz) before being digitized at a rate of 20 kHz.

Each subject completed a total of nine training sessions of isometric wrist extension within three weeks (3 days/week). They completed the training protocols by application of an inflated cuff (540 mm long x 68 mm width) (SAGA Fitness, SAGA Fitness International, Australia) on the muscle belly of the biceps brachii of the non-dominant limb (Fig. 1(A)). The occlusion pressure was set at 70% of the systolic blood pressure (SBP) after predetermination of the SBP with an electronic sphygmomanometer (466 mm long x 45 mm width, Model: HEM-121, Omron, Healthcare Co., Ltd. Kyoto, Japan) in the beginning of the training session. Cuff occlusion was applied for six minutes before the resistance training. During the training session, participants in the BFR-HL group were trained with four sets of 10 repetitions of brief isometric wrist extension of 75% MVC at a rate of 1 Hz guided by a metronome. The force exertion and the target force were visually guided on the computer monitor. In the BFR-LL group, the participants were trained with eight sets of 15 repetitions of isometric wrist extension of 25% MVC at a rate of 1 Hz. To prevent exhaustion, 30s rest periods were interleaved between contraction sets for the BFR-HL and BFR-LL groups. The total training volume of a training session (sets × repetition × intensity) for the two groups was identical (BFR_HL: 4 sets* 10 repetitions * 75% MVC; BFR_LL: 8 sets* 15 repetitions * 25% MVC).

### Data analysis and signal processing

The strength gain following the BFR training was defined as normalized change in MVC value ((post-test -pre-test)/pre-test)*100%. The force signal during force-tracking initially underwent low-pass filtering (cutoff frequency, 6 Hz) to eliminate high-frequency noise, with a focus on the effects of visuomotor processes on force output in the 0- to 4-Hz band [23]. In both the pre-test and post-test, only force data within the window of interest were analyzed. Force fluctuations were characterized as force output after removal of the linear trend. In terms of root mean square (RMS), the size of force fluctuations represents the capacity for force precision control. Additionally, we calculated the approximate entropy (ApEn) of force fluctuations [24, 25]. Force fluctuation signals were down-sampled to 100 Hz before ApEn calculation with the following parametric settings: dimensions, 2; lag, 1; radius, 0.2 times the variance of the signal. ApEn, which ranges from 0 to 2, quantifies the regularity of physiological time series. Lower ApEn values indicate more regular force fluctuations. Higher values signify greater irregularity, reflecting a more automatic control with less attentive regulation of force employed [26].

Post-decomposition analysis of the surface EMG was performed in EMG Works version 4.3 (Delsys Inc.), based on an artificial intelligence computation algorithm [27, 28] for differentiating superimposed action potentials in surface EMG signals into motor unit action potential (MUAP) waveforms and discharge events of motor units (Fig. 1(B)). The accuracy of the decomposition algorithms was cross-validated using the decompose–synthesize–decompose–compare (DSDC) approach [27–29] and two-source methods [30]. Only MUs with a decomposition accuracy exceeding 90% were selected for further analysis. The inter-spike intervals (ISI) of these identified MUs within the window of interest were averaged to calculate the mean inter-spike interval (M_ISI). Discharge variability of a MU in the window of interest was represented by the coefficient of variation of the inter-spike intervals (CV_ISI). During the ramp-up phase (0–75% MVC), the recruitment thresholds (Rec_th) of motor units were identified. These thresholds corresponded to the normalized force level (% MVC) at the moment when the first firing of the motor unit occurred (Fig. 1(B)) [26]. To characterize the common synaptic inputs to the pool of motor neurons, classical cross-correlation was employed [31, 32]. The smoothed time-varying firing rate signal of a MU was estimated by convoluting the impulse trains of MUs with a Hanning window (window length: 400 ms). Cross-correlation of the smoothed firing rate of MU pairs was then calculated, resulting in a peak value that occurred within 100 ms in the center of the cross-correlation plot. For variance stabilization, the cross-correlation plots of smoothed discharge rates were remapped using Fisher’s z transformation [32]. The normalized correlation peak of a MU pair was defined as the common drive index (CDI).

### Statistical analysis

In terms of MVC, differences in the force generation capacity between pre-test and post-test for the BFR-LL and BFR-HL groups were examined by paired t statistics on an individual basis. Group differences in normalized change in MVC value ((post-test-pre-test)/pre-test) between the BFR-LL and BFR-HL groups following BFR training was compared with independent t statistics and Cohen’s d. To examine variations in tracking performance due to BFR training, paired t-test were used to contrast tracking performance (FF_RMS_ and FF_ApEn_) between the pre-test and post-test for the BFR-HL and BFR-LL groups. Group differences in normalized changes in tracking performance (FF_RMS_ and FF_ApEn_) between the BFR-LL and BFR-HL groups were examined with independent t statistics and Cohen’s d. In case of violations of data homogeneity examined with Levene’s test, Welch’s t-test was used to correct the problem by accurate approximation of the degrees of freedom under the assumption of unequal variances. Considering variations in the number of motor units (MUs) resulting from decomposition across participants, motor unit variables before and after BFR training were compared based on pooled MU data. For relatively large sample sizes, permutation Hotelling’s T^2^ test and post-hoc permutation paired t-tests were applied to contrast differences in Rec_th, mean ISI, and CV_ISI of pooled motor units between pre-test and post-test for the two groups. The influence of BFR training on CDI for the BFR-HL and BFR-LL groups was examined using permutation paired t-tests. Data were analyzed in SPSS version 22.0 and MATLAB 2018 (MathWorks Inc.). Mean values ± standard deviations are reported in the text, figures, and tables.

## Results

Table 1 presents the means and standard deviations of MVC for both groups before and after BFR resistance training. Paired t-statistics revealed consistent increases in MVC following training in both the BFR-HL (*t*_*13*_ = -5.655, *p* < 0.001; Cohen’s d = 1.511) and BFR-LL groups (*t*_*13*_ = -2.850, *p* = 0.014; Cohen’s d = 0.762). The BFR-HL group exhibited a greater strength gain in MVC than that of the BFR-LL group (BFR-HL: 26.96 ± 16.33%, BFR-LL: 11.16 ± 15.34%)(*t*_*26*_ = 2.473, *p* = 0.020; and Cohen’s d = 0.935)(Table 1).

**Table 1:** The contrast of maximal voluntary contraction (MVC) between the high-load (BFR-HL) and low-load (BFR_LL) groups

MVC (NT)	Pre-test	Post-test	Normalized Change	Statistics
BFR-HL	89.49 ± 29.02	110.97 ± 34.90^†††^	26.96 ± 16.33%	*t*_*26*_ = 2.473, *p* = 0.020
BFR-LL	92.67 ± 31.72	100.85 ± 32.70^†^	11.16 ± 15.34%	Cohen’s d = 0.935

MVC: maximal voluntary contraction; BFR-HL: high-load group; BFR-LL: low-load group

^†^: Post-test > Pre-test, *p* < 0.05; ^†††^: Post-test > Pre-test, *p* < 0.001

Cohen’s d = 1.511

Cohen’s d = 0.762

All participants in the BFR-HL and BFR-LL groups were able to maintain target force during force tracking for the entire duration of each trial. Table 2 displays the means and standard deviations of force tracking performance for both groups before and after BFR resistance training. The results of paired t-tests indicated that the size of force fluctuations (FF_RMS_) in the BFR-LL group decreased after training (*t*_*13*_ = 3.474, *p* = 0.004; Cohen’s d = 0.924), contrasting with the insignificant trend observed in the BFR-HL group (*t*_*13*_ = 0.501, *p* = 0.625; Cohen’s d = 0.019). Additionally, the normalized change in FF_RMS_ varied by group, with the BFR-LL group exhibiting a more negative normalized change compared to the BFR-HL group (*t*_*19.53*_*= 2.078*, *p* *= 0.050*; Cohen’s d = 0.785). For the approximate entropy of force fluctuations (FF_ApEn_), the results of paired t-tests indicated that the FF_ApEn_ in the BFR-LL group potentiated after training (*t*_*13*_ = -4.880, *p* < 0.001), contrasting with the trend observed in the BFR-HL group (*t*_*13*_ = -1.163, *p* = 0.266; Cohen’s d = 1.291). The normalized change in FF_ApEn_ was marginally dependent on group (*t*_*26*_ = -1.749, *p* = 0.092; Cohen’s d = 0.661), with the BFR-LL group demonstrating an increasing trend of normalized change compared to the BFR-HL group.

**Table 2:** The contrast of force fluctuation properties during force tracking between the high-load and low-load groups

	MVC (NT)	Pre-test	Post-test	Normalized Change	Statistics
FF_RMS_(%MVC)	BFR-HL	2.09 ± 0.79	1.94 ± 0.85	0.00 ± 42.52%	*t*_*19.53*_ = 2.078, *p* = 0.050
	BFR-LL	1.94 ± 0.73	1.29 ± 0.32^**^	-27.51 ± 22.05%	Cohen’s d = 0.785
FF_ApEn_	BFR-HL	0.285 ± 0.068	0.305 ± 0.065	10.08 ± 26.21%	*t*_*26*_ = -1.749, *p* = 0.092
	BFR-LL	0.273 ± 0.059	0.337 ± 0.056^†††^	26.83 ± 22.50%	Cohen’s d = 0.661

MVC: maximal voluntary contraction; BFR-HL: high-load group; BFR-LL: low-load group

FF_RMS_: root mean square of force fluctuations; FF_ApEn_: approximate entropy of force fluctuations

^**^: Post-test < Pre-test, *p* < 0.005; ^†††^: Post-test > Pre-test, *p* < 0.001

**Fig. 2:**
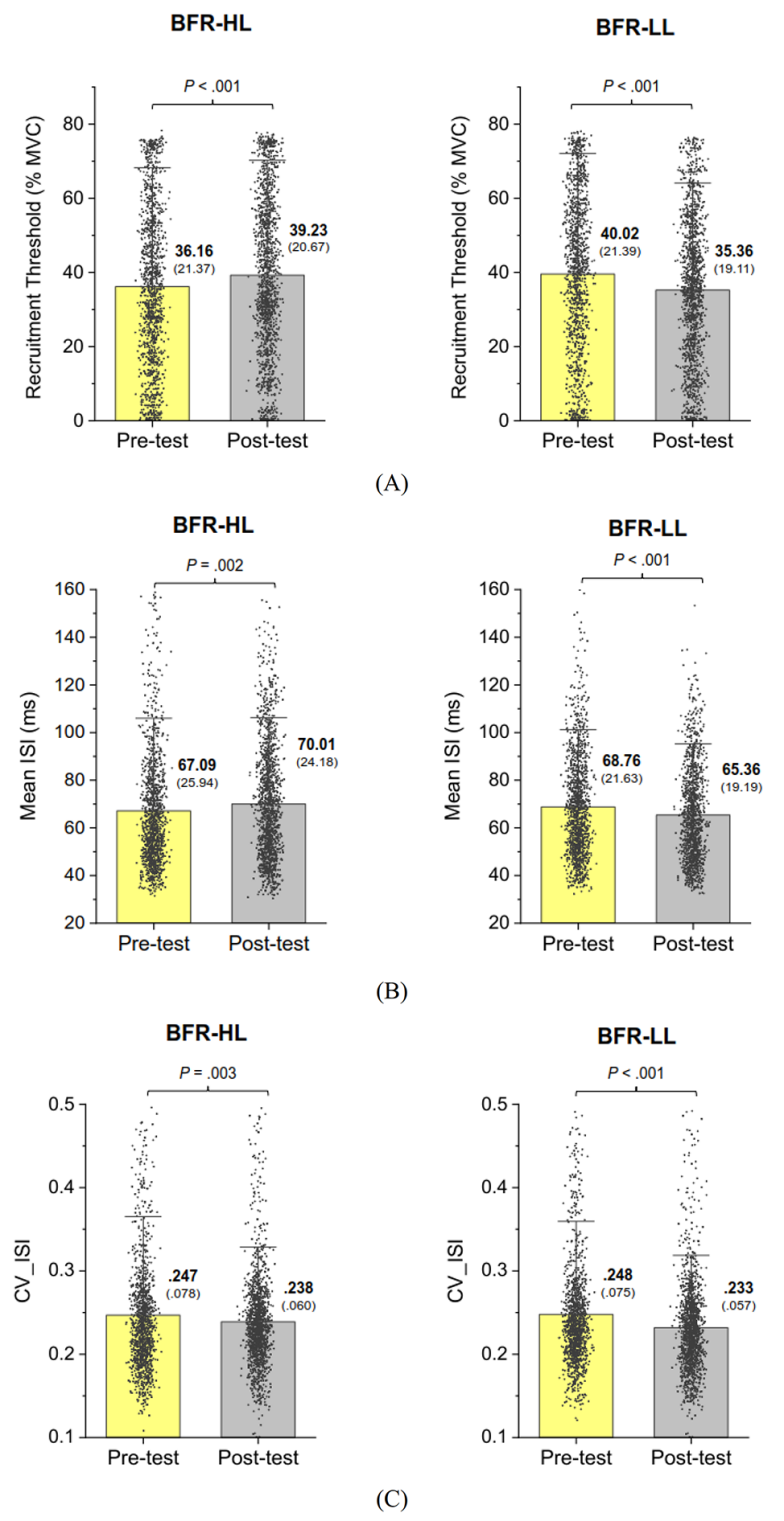
The contrasts of motor unit variables between the pre-test and post-test. The mean and standard deviations of recruitment threshold (**A**), mean inter-spike interval (M_ISI) (**B**), and coefficient of variation (CV-ISI) (**C**) of inter-spike interval in the pre-test and post-test for the BFR-HL and BFR-LL groups. Each small dot represents variables of an individual motor unit in the BFR-HL or BFR-LL group

**Fig. 3:**
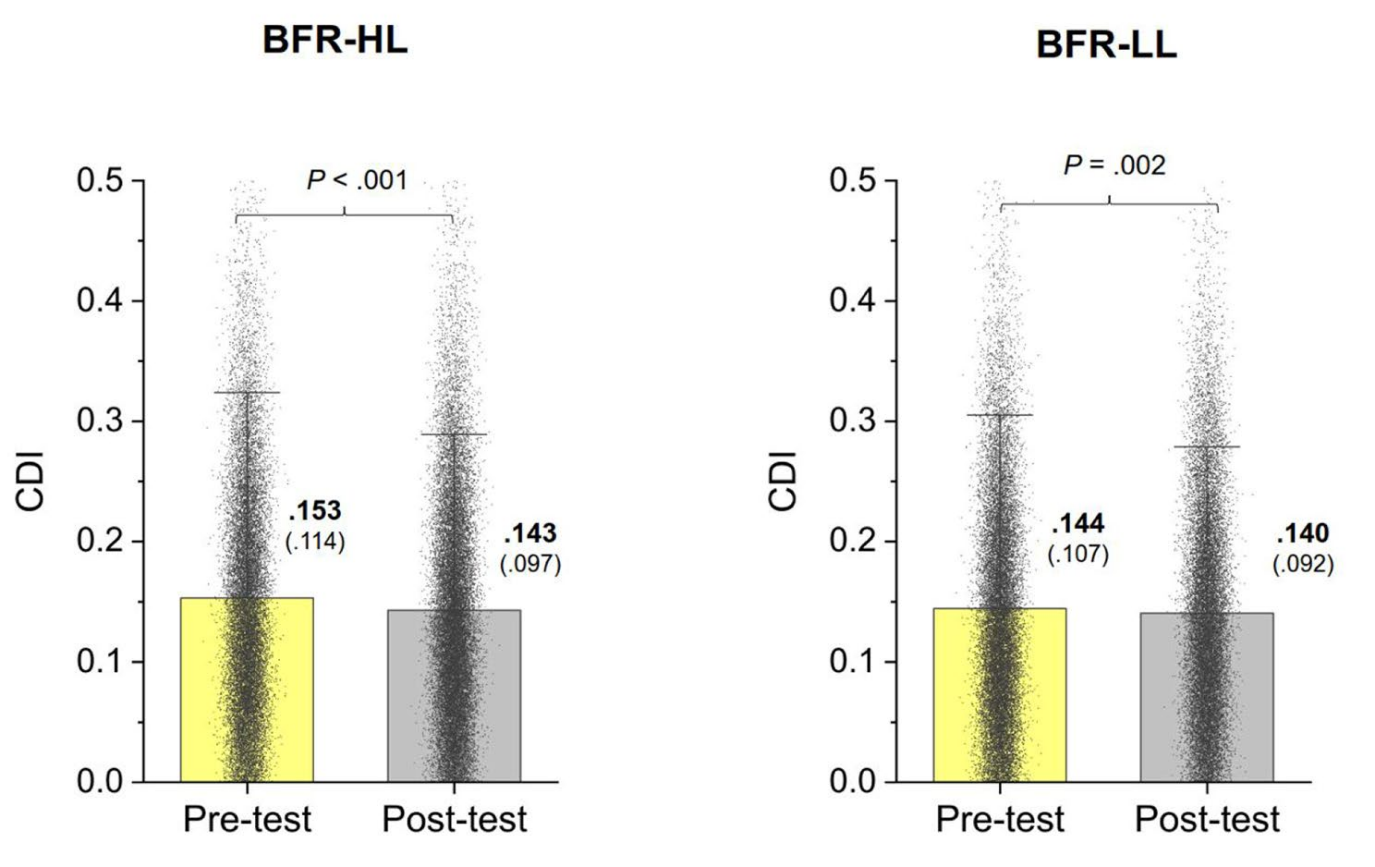
The contrasts of common drive index (CDI) between the pre-test and post-test. Each small dot represents a CDI value of a motor unit pair in the BFR-HL or BFR-LL group

The numbers of MUs decomposed from multi-electrode EMG from all participants across three trials for the BFR-HL group in the pre-test and post-test were 988 and 1131, respectively. The numbers of decomposed MUs for the BFR-LL group in the pre-test and post-test were 976 and 987, respectively. Figure 2(A)-(C) illustrate the training-related changes in MU variables during force-tracking for the BFR-HL and BFR-LL groups. Permutation Hotelling’s T^2^ test results indicated that MU variables differed significantly before and after BFR resistance training in both groups (BFR-HL: *p* < 0.001; BFR-LL: *p* < 0.001). Post-hoc analysis with permutation t statistics revealed that in the post-test, the MU recruitment threshold (Rec_th) of the BFR-HL group increased (*p* < 0.001), while the mean MU Rec_th of the BFR-LL group decreased (*p* < 0.001), relative to those in the pre-test (Fig. 2(A)). For the BFR-HL group, mean ISI was significantly larger in the post-test than in the pre-test (*p* = 0.002). Conversely, mean ISI was significantly smaller in the post-test than in the pre-test for the BFR-LL group (*p* < 0.001) (Fig. 2(B)). Both the BFR-HL (*p* = 0.003) and BFR-LL (*p* < 0.001) groups demonstrated significant post-test declines in CV_ISI. Figure 3 illustrates the contrast in CDI between the pre-test and post-test for the BFR-HL and BFR-LL groups. The two groups displayed parallel decreasing trends in CDI with BFR resistance training, indicating a lower level of CDI in the post-test (*p* ≤ 0.002).

## Discussion

In a volume-matched condition, both BFR-HL and BFR-LL consistently led to improvements in maximal voluntary contraction of wrist extension. However, BFR-HL training resulted in superior improvement in maximal voluntary contraction compared to BFR-LL training. Notably, only BFR-LL training improved force precision control of wrist extension during force tracking, as evidenced by smaller force fluctuations with lower regularity in the post-test; BFR-HL training did not show such improvements. The load-dependent differences in force precision control during force-tracking were associated with variations in adaptive changes in motor unit (MU) activation strategies. BFR-LL training favored the recruitment of MUs with lower recruitment thresholds and higher firing rates. In contrast, BFR-HL training tended to activate MUs with higher recruitment thresholds and lower firing rates. Both BFR training protocols reduced the discharge variability of MUs and MU synchrony within a MU pair. These findings highlight the distinct effects of BFR training on neuromuscular adaptations and force control, with load playing a significant role in determining the outcomes.

### Differential force behaviors with training load for BFR protocols

The present results regarding the superior force generation capacity following BFR-HL training were inconsistent with previous studies [8, 33], where the total training volume was not exactly matched. The ongoing debate concerning strength improvements in blood flow restriction (BFR) training of varying loads persists, potentially due to a lack of comparability in total strength volume between BFR-HL training and BFR-LL training. Theoretically, BFR-HL training is more susceptible to excessive metabolic accumulation and the early development of fatigue responses [34, 35]. With matched training volumes, this study revealed better post-test strength gain in MVC after 3 weeks of BFR-HL training (26.96 ± 16.33%) compared to BFR-LL training (11.16 ± 15.34%)(Table 1). Interestingly, BFR-LL training not only enhanced post-test MVC but also improved force precision control, as evidenced by a significant reduction in the sizes of post-test force fluctuations (Table 2). The improvement in force scaling after BFR-LL training was also associated with an increasing trend of the complexity of force fluctuations (or enhanced ApEn) (Table 2). The additive accuracy mechanism proposes that force fluctuations (or intermittency) serve as a means to correct tracking deviations [36, 37]. In this context, the adaptive increase in force fluctuation complexity might be interpreted as an improved automatic response for tracking deviation correction that requires less information processing and attentive control following BFR-LL training [38]. In parallel, young adults consistently demonstrate higher movement complexity than aged individuals [39], who experience a loss of behavioral repertoire due to degenerative changes. The distinct performance benefits observed for BFR-HL and BFR-LL training may not be attributed to metabolic stress, as phosphocreatine depletion and intramuscular pH decrease were similar for different BFR training protocols with the same total work volume [40]. Some non-metabolic factors, such as MU recruitment and cellular swelling, may account for the mechanic differences. The present findings have practical implications for the use of BFR resistance training. When training volume is appropriate, power-type athletes may benefit more from BFR-HL training for strength improvement. Despite yielding less strength gain, BFR-LL training enhances force precision control, which is crucial for pistol shooters, surgeons, and rehabilitative patients who need to regain muscle strength and the ability to control their movements precisely to avoid action errors or re-injury.

### Load-dependent adaptive changes in motor unit activation strategy

This study revealed that adaptive changes in MU activation strategies depended on the training loads in the BFR protocols, as illustrated in Fig. 2(A)-(C). BFR could induce metabolic and/or mechanical stress, which can influence how motor units behave and selective hypertrophy muscle fibers [41–43] for low-load resistance exercise. Fatela et al. (2019) reported an immediate effect of BFR that shifts MU recruitment pattern with a more negative regression slope between MU recruitment threshold and firing rate [44]. Hence, BFR not only recruits additional MUs with higher thresholds but also facilitates the firing of MUs with lower thresholds, compared to non-BFR conditions. The present study further indicates distinct adaptive changes in MUs in response to resistance training with different loads under BFR. BFR-LL training led to a decrease in the mean recruitment threshold of MUs, with shorter inter-spike intervals during post-test force-tracking, whereas an opposite trend was noted for BFR-HL training (Fig. 2(A), (B)). In the context of the reduction in recruitment threshold after BFR-LL training, the CNS can more readily access MUs with lower thresholds and discharge at higher rates during post-test force-tracking, emphasizing endurance-related neural adaptations. In contrast, BFR-HL training involves the recruitment of higher-thresholds MUs with longer inter-spike interval during post-test force-tracking. The scenarios suggest an expansion of the MU recruitment pool for effective force generation after BFR-HL training, aligning with the prediction of the onion-skin motor unit control scheme [45].

Although a direct causal link between force precision and MU behaviors remains to be firmly established, the observed adaptations in MU recruitment strategy and rate coding have the potential to influence load-dependent differences in force precision control following BFR training. After BFR-LL training, the increase in discharge rate of lower-threshold MUs was likely to reduce the size of force fluctuations (Table 2), as shorter inter-spike intervals facilitate fusion of twitch forces generated by these MUs. Additionally, post-test reductions in discharge variability (Fig. 2(C)) and MU synchrony (Fig. 3) were also beneficial for improving force steadiness, as variability in motor unit discharge rates can largely account for fluctuations in motor output during isometric contraction [46, 47]. It is noteworthy that BFR-LL training could lead to reduced discharge variability and force fluctuations at higher exertion levels (such as 75% MVC) in this study, a phenomenon not observed with short-term traditional low-intensity endurance training. Traditional low-load resistance training might indeed reduce discharge variability at much lower exertion levels (20% MVC) [48], underscoring the additional benefits of BFR during low-intensity resistance training. On the other hand, BFR-HL training resulted in the additional recruitment of higher-threshold MUs (Fig. 2(B)), which are more effective in force development. However, the decrease in discharge rate of the MUs (Fig. 2(B)) was not conducive to force precision control (Table 2), as it led to ineffective fusion of twitch forces for longer inter-spike intervals. Apparently, the detrimental effect on force steadiness cannot be compensated by concurrent decrease in discharge variability following BFR-HL training (Fig. 2(C)).

### Methodological considerations

There were some methodological concerns in this study. First, this study did not replicate previous works examining MU adaptations to resistance training of varying loads without BFR by including a non-BFR control group, as the primary focus of this study was to delineate the training effects of BFR with HL and LL protocols. While this study addressed MU remodeling related to training under BFR, we cannot dismiss the possibility of load-dependent MU adaptation occurring with traditional resistance training of matched volume without BFR. A review article by Duchateau et al. (2006) [49] concluded that non-BFR strength training with heavy loads (70% of maximum) did not improve either steadiness or discharge rate variability. Conversely, light-load training was effective in reducing discharge and force variability in the index finger for older adults [50]. Nonetheless, accumulating evidences suggest that recruitment strategies and discharge behaviors of motor units differs with BFR application [22, 48, 51]. BFR tends to increase muscle excitation during a single low-load [48] and high-load [51] contraction, by activating MUs with higher recruitment thresholds. For motor units of similar size, they discharged at higher rates following BFR training [22, 48]. Expanding on prior research, the current study fills knowledge gaps by comparing MU adaptations between high-load and low-load training under BFR. Second, the same motor units were not precisely tracked across the training intervention, even though electrode positioning was performed carefully in both the pre-test and post-test. As a result, the study pooled data from recorded motor units, approximately 1000 MUs, assuming no distribution differences in recruitment thresholds of these MUs before and after BFR training. The research focus was primarily on global changes in the discharge behaviors of a population of motor units rather than individual units. Additionally, it is important to note that this study focused on contrasting the short-term training benefits of BFR-HL and BFR-LL training. Future research is needed to differentiate the long-term training benefits between these two training regimens. The use of various BFR training protocols (such as occlusion pressure), the design characteristics of BFR devices (such as cuff material and width), and exercise protocols (such as concentric or eccentric exercise) may influence the specific adaptive changes in motor unit and force behaviors observed with each type of resistance training. Finally, the relatively small sample size in this study may have limited data homogeneity and statistical power to generalize the findings of this research. Despite these concerns, our findings still provide novel evidence of the differential impacts on force regulation following BFR-HL and BFR-LL training.

## Conclusion

This study is the first to report differential training benefits and the underlying motor unit physiology for short-term BFR-HL and BFR-LL training with matched training volumes. Specifically, BFR-HL training leads to superior strength gains compared with BFR-LL training. However, BFR-LL training is associated with improved force precision control, a benefit not observed in BFR-HL training. The variations in the capacity for force scaling observed with BFR training associated with differing motor unit activation strategies. Force scaling following BFR-HL training tends to recruit MUs with higher thresholds. In contrast, improved force scaling after BFR-LL training involves adaptive changes in rate coding by increasing the discharge rate of MUs with lower thresholds.

## Data Availability

No datasets were generated or analysed during the current study.

## Abbreviations

ApEn: Approximate entropy
BFR: Blood flow restrictions
CDI: Common drive index
CV_ISI: Coefficient of variation of the inter-spike intervals
DSDC: Decompose–synthesize–decompose–compare
EMG: Electromyography
MAPK: Mitogen-activated protein kinase
mTOR: Mammalian target of rapamycin
MU: Motor unit
MUAP: Motor unit action potential
MVC: Maximal voluntary contraction
HL: High load
ISI: Inter-spike intervals
LD: Linear deviation
IGF-1: Insulin-like growth factor 1
LL: Low load
M_ISI: Mean inter-spike interval
Rec_th: Recruitment thresholds
RM: Repetition maximum
ROS: Reactive oxygen species
SBP: Systolic blood pressure
